# Calculated initial parenteral treatment of bacterial infections: Bacterial endocarditis

**DOI:** 10.3205/id000052

**Published:** 2020-03-26

**Authors:** Pascal M. Dohmen, Klaus Friedrich Bodmann, Wolfgang Graninger, Pramod Shah, Florian Thallhammer

**Affiliations:** 1 Klinik und Poliklinik für Herzchirurgie, Universitätsmedizin Rostock, Germany; 2 Klinik für Internistische Intensiv- und Notfallmedizin und Klinische Infektiologie, Klinikum Barnim GmbH, Werner Forßmann Krankenhaus, Eberswalde, Germany; 3 Vienna, Austria; 4 Frankfurt am Main, Germany; 5 Klinische Abteilung für Infektiologie und Tropenmedizin, Medizinische Universität Wien, Vienna, Austria

## Abstract

This is the twelfth chapter of the guideline “Calculated initial parenteral treatment of bacterial infections in adults – update 2018” in the 2^nd^ updated version. The German guideline by the Paul-Ehrlich-Gesellschaft für Chemotherapie e.V. (PEG) has been translated to address an international audience.

The bacterial endocarditis is characterised by a constant incidence but a shift in the patient population due to the use of prosthetic heart valves and foreign materials like pacemakers and the increasing application of invasive medical procedures. This is linked to a change in the predominant infecting organisms towards staphylococci. This chapter gives recommendations for the interdisciplinary management of infective endocarditis from the diagnostic workup over prevention to therapy with a focus on antibiotic therapy.

## Zusammenfassung

Dies ist das zwölfte Kapitel der von der Paul-Ehrlich-Gesellschaft für Chemotherapie e.V. (PEG) herausgegebenen S2k Leitlinie „Kalkulierte parenterale Initialtherapie bakterieller Erkrankungen bei Erwachsenen – Update 2018“ in der 2. aktualisierten Fassung.

Die bakterielle Endokarditis ist gekennzeichnet durch eine gleichbleibende Inzidenz, aber eine Veränderung in der betroffenen Patientenpopulation durch den Einsatz von Klappenprothesen, Fremdmaterialien wie Schrittmachern und den zunehmenden Einsatz invasiver medizinischer Maßnahmen. Damit verbunden ist eine Veränderung des Erregerspektrums hin zu Staphylokokken auch bei der Nativklappen-Endokarditis. Dieses Kapitel gibt Empfehlungen für das interdsiziplinäre Management der bakteriellen Endokarditis von der Diagnostik über die Prävention zur Therapie mit dem Fokus auf der Antibiotika-Therapie und evaluiert die aktuellen Empfehlungen der ESC aus deutscher Sicht.

## Introduction

Bacterial endocarditis was first mentioned in the 17^th^ century by Morgagni [1]. The aetiology of this infection was only described 100 years later by Rokitansky. It identified bacteria which existed within embolized vegetation [2]. Today, bacterial endocarditis continues to be a disease with significant morbidity and mortality, although antimicrobial and surgical intervention has improved substantially [3]. The incidence of bacterial endocarditis is 1 case per 1,000 hospitalizations in internal medicine [4].

The annual incidence of bacterial endocarditis of native heart valves in Europe is around 3 cases per 100,000 inhabitants [5]. As several studies have shown, the incidence of bacterial endocarditis has remained almost constant in recent decades [6]; this is due to a redistribution of predisposing factors. A systematic review from several Western European countries showed a significant increase in patients with prosthetic heart endocarditis and endocarditis due to degenerative heart valve disease or intravenous drug abuse [7]. The increase in invasive medical procedures has increased the number of bacteremias and thus the proportion of bacterial healthcare-associated endocarditis [8]. This increase of up to 34% in all endocarditis cases [9] also results in the average age increasing 10–15 years in recent years [10], [11].

## The hospital setting

A major problem is the long delay between the first occurrence of unrecognized symptoms and the onset of microbial invasion of the endocardium in intravascularly implanted foreign body materials such as heart valve prostheses, pacemaker electrodes, micra cardiocapsules, occluders, and artificial hearts. In Germany in particular, culture-negative endocarditis is common, further complicating definitive diagnosis. The resulting delay to initiating of adequate antibiotic treatment can significantly increase morbidity and mortality [12]. Classic guiding symptoms are often difficult to assess, such as a heart murmur in a patient who has not previously been examined by a cardiologist or non-specific symptoms such as sub-febrile temperature, fever, night sweats, weight loss, loss of appetite, general fatigue, myalgias and arthralgias. Not infrequently, initial symptoms are already signs of incipient complications, such as progressive stress dyspnoea and subsequent orthopnea, which indicates valve destruction with loss of valve function due to a heart valve insufficiency with significant volume overload. Signs of central septic embolization may be the first symptoms of neurological deficits. Right-sided endocarditis may cause symptoms of pulmonary embolization. Furthermore, peripheral micro- or macroemboli may be present in combination with immunological phenomena, for example Osler nodules, Jayneway lesions and splinter hemorrhages.

If risk factors are present, such as intravenous drug abuse or in patients with intravascular implants made from foreign materials, when carrying out a differential diagnosis bacterial endocarditis must always be a consideration, even with non-specific symptoms. For clinical evaluation, the modified Duke criteria should always be used in these patients [13] and, if appropriate, a team of endocarditis experts from a nearby hospital providing maximal medical care should be included [14].

## Prevention

Since bacterial endocarditis is a dangerous and difficult-to-treat bacterial infection, endocarditis prophylaxis has been used in risk patients for over 50 years. In 2002, the indications for such prophylactic care were redefined as part of a risk-benefit analysis [15]. These results have been successively incorporated into all international guidelines in recent years [16], [17], [18], [19], [20].

The 2007 American Heart Association guidelines [16] implemented the new indications for endocarditis prophylaxis [21]. The decisive factor was that nowadays such guidance relies exclusively on evidence-based data. Previously the guidelines were based on animal studies and expert opinions, rather than prospective, randomized and double-blind studies as one would expect today. Due to the changes to the indication of endocarditis prophylaxis, it is now only performed in patients at the highest risk of bacterial endocarditis. 

In 2008, the National Institute for Health and Care Excellence (NICE) recommended refraining from endocarditis prophylaxis for both dental and non-dental patients [22]. An epidemiological study in the UK conducted following this recommendation showed an increase in the number of bacterial endocarditis cases after the NICE guidelines came into force in 2008 [23]. A survey conducted in the UK in 2012 found that the majority of cardiologists and cardiac surgeons advocated the need for antibiotic prophylaxis in patients with heart valve prostheses [24]. Another UK study showed a significant increase in the incidence of bacterial endocarditis in both high-risk and low-risk patients, so the introduction of NICE guidelines in 2008 cannot be taken as the sole cause of this fact [8].

## Interdisciplinary care

The interdisciplinary treatment of patients with bacterial endocarditis begins with good communication between the referring GP and the hospital doctor in charge of admission. The interdisciplinary approach must be continued within the hospital. 

The prerequisite for including a patient in the SYNTAX multicentre randomized study, which looked at patients with coronary heart disease, was interdisciplinary collaboration between cardiologists and cardiac surgeons [25]. This concept of a multidisciplinary cardiac team to determine the course of treatment has been adopted for highest-risk patients suffering from aortic valve disease. In such cases, an interdisciplinary team determines which patient will receive conventional surgical aortic valve replacement and which patient will be sent for transcatheter aortic valve implantation (TAVI). This team, consisting of cardiologists and cardiac surgeons, has been expanded to include an anesthesiologist [26]. This concept today is an integral part of European guidelines. 

Up until now, cardiology guidelines for treating patients with bacterial endocarditis required an interdisciplinary team of physicians including cardiologists, microbiologists, infectiologists and cardiac surgeons. Due to the complexity of this condition, there was a call for additional experts to be consulted, such as neurologists, neurosurgeons, radiologists, nuclear medicine specialists and anesthesiologists, as well as clinical pharmacologists and in the case of congenital disorders, pediatric cardiologists and pediatric cardiac surgeons. The introduction of these measures has significantly reduced the mortality of endocarditis from 18.5% to 8.2% in a French study [27]. The survival of patients with bacterial endocarditis has been significantly improved elsewhere too by adopting this concept [28]. In 2014, these guidelines were also incorporated into the Guidelines for the Treatment of Patients with Heart Valve Disease [19] by the American Heart Association/American College of Cardiology with a class 1b recommendation. The European Society of Cardiology complied with these guidelines when preparing the ESC guidelines for the treatment of patients with bacterial endocarditis published in 2015 [29].

## Imaging

The core diagnostic imaging procedure in transthoracic and, above all, in transesophageal examinations is echocardiography. This examination method is of great importance for pre-operative diagnosis, for pre-operative monitoring, for evaluating the outcome of surgery as well as for post-operative follow-up. However, due to the complexity of today’s endocarditis with an increase in healthcare-associated bacterial endocarditis, additional imaging techniques are needed, such as magnetic resonance imaging, multislice computed tomography or nuclear medical techniques such as single-photon emission tomography (SPECT) and ^18^F-fluorodeoxyglucose (FDG) positron emission tomography. The necessity of echocardiography as a core examination has already been pointed out. It occupies a key position in terms of diagnosis and assessment. Transesophageal echocardiography is significantly superior in sensitivity to transthoracic echocardiography [30], [31], especially when tricuspid and pulmonary valves in implanted foreign body materials are concerned. However, with respect to florid endocarditis, the specificity of echocardiography is limited [32]. In the presence of clinically suspicious factors however, for example an unclear fever with positive blood cultures in which typical pathogens have been detected, clarification including an echocardiographic examination is absolutely necessary. If active endocarditis is detected, weekly echocardiographic follow-up is useful. 

Advanced imaging procedures as described above are of increasing importance in view of the percentage increase in positive blood cultures due to the increasing implementation of foreign materials in patients through invasive procedures and the more and more frequently performed less invasive TAVIs. Studies have shown, for example, that cerebral septic embolisms occur in 33% of patients with endocarditis without clinical symptoms [33]. This fact only became clear through the use of advanced imaging methods. 

## Pathogen detection

Proof of the causative pathogen is crucial for rapid and targeted treatment. Correctly obtaining at least three blood culture pairs (aerobic and anaerobic) at different times via peripheral venipuncture, with strict adherence to the aseptic conditions for venipuncture and removal of the sample, is of paramount importance. Venous blood cultures are superior to arterial cultures due to fluid dynamic factors. 

Because bacterial endocarditis constitutes a continuous bacteremia, blood cultures can be obtained at any time without the need for taking body temperature into account. It is important to note that fever peaks, caused by decomposition of pathogens, represent an unfavorable time for obtaining blood cultures because pathogen density in the blood is particularly low at this point in time. The obtained blood cultures must be immediately forwarded to a microbiological lab. 

In up to 30% of cases, pathogens cannot be identified in blood cultures, often due to antibiotic treatment before cultures are obtained. In patients with endocarditis and negative blood cultures who are not critically ill, antibiotic treatment should be interrupted for a period of 48 hours in order to improve conditions for detecting pathogens in a subsequent blood culture. Another cause of negative blood cultures may be the presence of difficult-to-cultivate pathogens such as *Bartonella* spp., *Coxiella* spp., *Mycoplasma* spp., *Chlamydia* spp., *Tropheryma* *whipplei*, fungi and HACEK group pathogens. The HACEK group includes *Haemophilus* spp., *Actinobacillus* *actinomycetemcomitans*, *Cardiobacterium* *hominis*, *Eikenella* *corrodens* and *Kingella* spp. It is important to inform the microbiological lab if endocarditis is suspected in order to ensure an adequate examination of the materials. Currently there are culture-independent techniques for the optimization of microbiological diagnostics through application of molecular biological and serological methods. Fluorescence in-situ hybridization (FISH) combines molecular biology and histological techniques, which may also be helpful in detecting pathogens. Biopsies of heart valves, heart valve prostheses or peripheral embolisms can also help to detect pathogens in addition to the polymerase chain reaction (PCR) based on whole blood or serum. Due to a lack of standardization, the relevance of these methods is still unclear.

In addition, the sonication of prostheses, i.e. cardiac pacemakers or heart valves, can increase the detection of pathogens by 30%. 

## Antimicrobial therapy

If the general condition of a patient is critical, empirical antibiotic treatment is started immediately but always after obtaining blood cultures. For native valve endocarditis and late endocarditis following heart valve replacement (>1 year after surgery or intervention), above all methicillin-sensitive *Staphylococcus* *aureus* strains (MSSA), various streptococcal species and *Enterococcus* *faecalis* should be expected (Table 1 (Tab. 1)). The microbiological results from the first infection episode may help inform further calculated antimicrobial treatment. However, in early endocarditis after heart valve replacement (<1 year after intervention or surgery) this is more likely to be associated with methicillin-resistant *Staphylococcus* *aureus* strains (MRSA), coagulase-negative staphylococci and also Gram-negative bacteria. Initial empirical treatment should, if possible, be modified once microbiological results are available. Treatment schemes for the most common endocarditis agents can be found in Table 2 (Tab. 2). Further detailed treatment recommendations as well as recommendations regarding complications indicating a need for surgical intervention and for the continuation of antibiotic treatment after surgical restoration can be found in the guidelines of the European Society of Cardiology [29].

## Evaluation of Recommendations for Antibiotic Treatment of Endocarditis by the European Society of Cardiology (ESC) 2015

Significant changes to the European guidelines include endocarditis prophylaxis, the interdisciplinary “endocarditis team”, the expansion of imaging modes and the results of a first randomized study on the surgical treatment of bacterial endocarditis.

The European guidelines continue to recommend antibiotic prophylaxis for risky interventions in patients with predisposing cardiac factors. Preliminary studies support this approach, as after the introduction of the NICE guidelines in 2008 [21] negative effects had been shown by the extreme restriction of the indication of antibiotic prophylaxis in the United Kingdom [23], [24].

The recommendation to empirically treat with ampicillin and flucloxacillin in native valve endocarditis does not result from study data but from the need to cover staphylococci, streptococci and *Enterococcus* *faecalis* in initial treatment. Because ampicillin/sulbactam is not available in all European countries for parenteral treatment and the combination of amoxicillin/clavulanic acid cannot be dosed adequately for toxicity reasons, the combination of aminopenicillin plus flucloxacillin was recommended. Since ampicillin/sulbactam is available for parenteral treatment in Germany, 4x 3 g of ampicillin/sulbactam would be an equivalent alternative, which has the advantage of having to deal with only one drug or a fixed combination.

## Surgical treatment and aftercare

The introduction of interdisciplinary endocarditis teams has shown that surgical treatment is an integral part of the overall treatment concept in bacterial endocarditis and should not only take place once antibiotic treatment has failed. The classic indications for surgical restoration which must be used in approximately 50% of all patients are the development of cardiac insufficiency, irreversible and infection-related destruction of cardiac structures and the appearance of emboli, newly-emergent AV blockages and endocarditis in heart valve prostheses, transcatheter heart valves, pacemaker systems and closure systems. The scores necessary for surgical risk stratification (EuroSCORE II and STS score) are of limited suitability in this, as they were primarily developed for coronary revascularization or degenerative heart valve replacement. For surgical intervention in bacterial endocarditis, special score systems have been developed, such as the De-Feo Score and the International Collaboration on Endocarditis Score (ICE Score). 

The advantage of early surgical restoration is that especially in native mitral or tricuspid valve endocarditis, heart valves can be operated on, possibly even in minimally invasive ways. Determining indication for early surgical restoration is often difficult and an important task for the “interdisciplinary endocarditis team”. A randomized study in a low-risk population with small case numbers showed that surgical intervention reduced the rate of embolism significantly within 48 hours up to 6 months after surgery. However, patient survival was unaffected. 

Patients who are provided with TAVI represent a special patient population. In order to be able to optimally treat these patients in the future, new strategies must be developed. 

The basic principle of surgical restoration is the complete removal of the infectious material and tissue in order to cure the patient’s endocarditis with adequate antibiotic treatment. In Austria, adequate antibiotic treatment is partly carried out as out-patient parenteral antibiotic therapy (OPAT). In Germany, this form of treatment has not yet made inroads.

Imaging techniques (such as echocardiography), blood cultures and serological examinations should serve to monitor treatment success and, if necessary, contribute to appropriate adjustment of therapeutic measures.

## Note

This is the twelfth chapter of the guideline “Calculated initial parenteral treatment of bacterial infections in adults – update 2018” in the 2^nd ^updated version. The German guideline by the Paul-Ehrlich-Gesellschaft für Chemotherapie e.V. (PEG) has been translated to address an international audience.

Following the publication of the 1^st^ version of the guideline in German, these dosage suggestions were updated by the working group (Table 1: Empiric therapy of culture-negative bacterial endocarditis with previous antibiotic therapy or until the blood culture results are obtained):

- gentamicin dosage in combination with ampicillin, ampicillin/sulbactam or vancomycin: 1x 3 mg/kg INSTEAD OF 3x 3mg/kg
- rifampicin dosage in combination with vancomycin: rifampicin 900 mg iv (2 SD) INSTEAD OF rifampicin 900 mg iv (3 SD)

## Competing interests

The authors declare that they have no competing interests.

## Figures and Tables

**Table 1:**
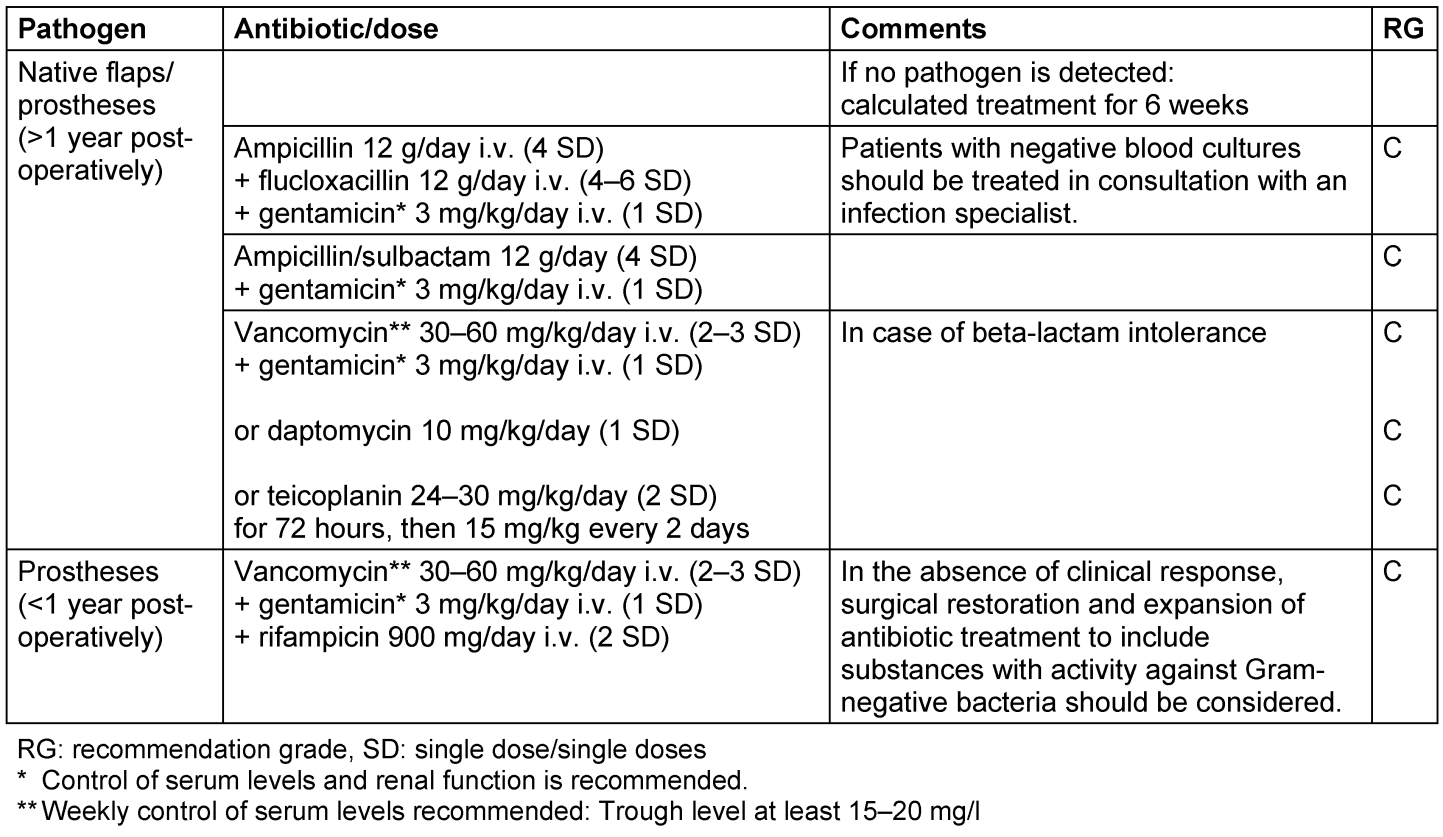
Empiric treatment of culture-negative bacterial endocarditis with prior antibiotic treatment or until blood culture results are ready. For culture-negative endocarditis without prior antibiotic treatment, an infection expert should be consulted.

**Table 2:**
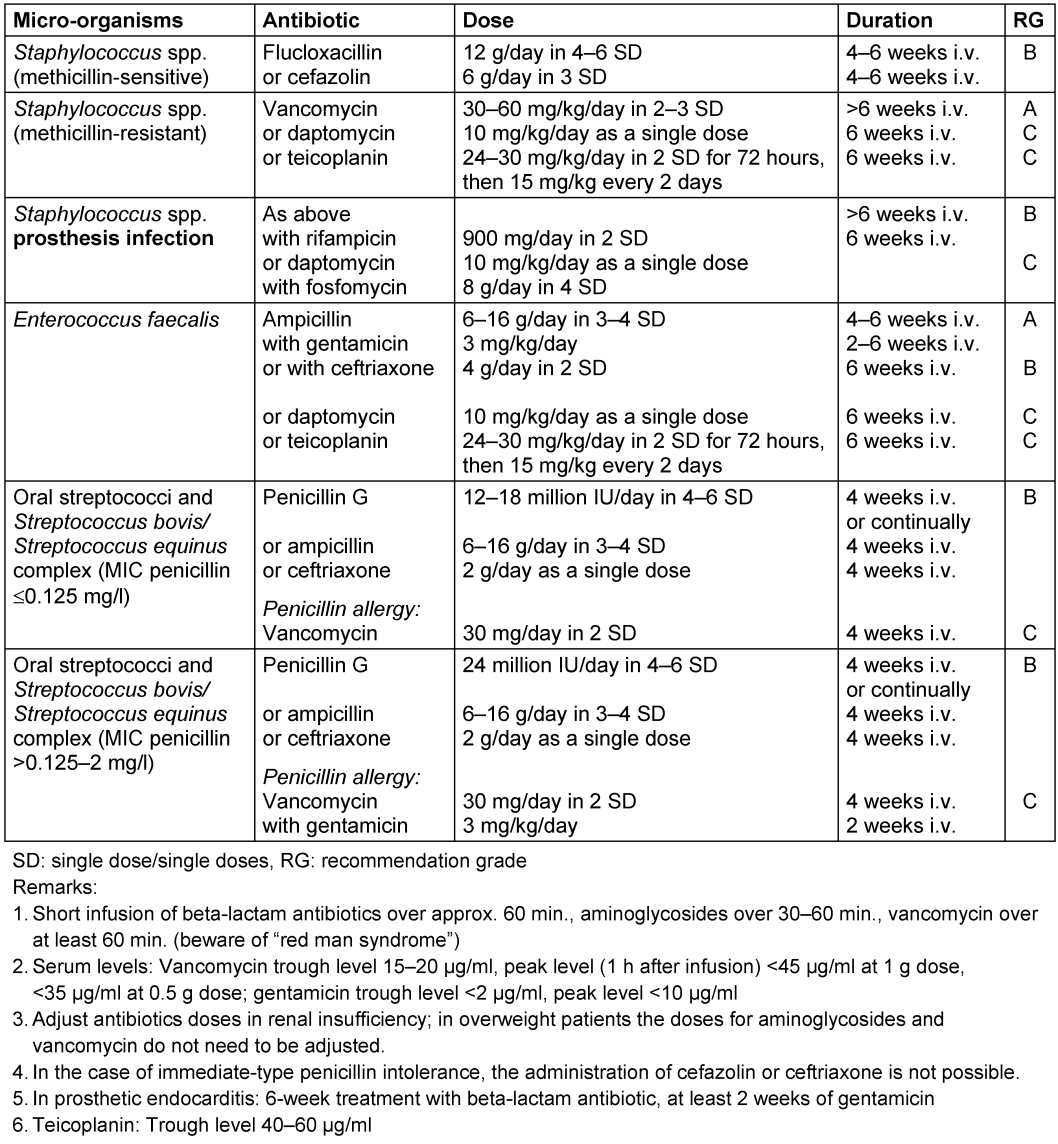
Overview of the common antibiotics for bacterial endocarditis
